# Importance of Glycosylation on Function of a Potassium Channel in Neuroblastoma Cells

**DOI:** 10.1371/journal.pone.0019317

**Published:** 2011-04-26

**Authors:** M. K. Hall, Tara A. Cartwright, Christa M. Fleming, Ruth A. Schwalbe

**Affiliations:** Department of Biochemistry and Molecular Biology, Brody School of Medicine at East Carolina University, Greenville, North Carolina, United States of America; Faculdade de Medicina, Universidade de São Paulo, Brazil

## Abstract

The Kv3.1 glycoprotein, a voltage-gated potassium channel, is expressed throughout the central nervous system. The role of *N*-glycans attached to the Kv3.1 glycoprotein on conducting and non-conducting functions of the Kv3.1 channel are quite limiting. Glycosylated (wild type), partially glycosylated (N220Q and N229Q), and unglycosylated (N220Q/N229Q) Kv3.1 proteins were expressed and characterized in a cultured neuronal-derived cell model, B35 neuroblastoma cells. Western blots, whole cell current recordings, and wound healing assays were employed to provide evidence that the conducting and non-conducting properties of the Kv3.1 channel were modified by *N*-glycans of the Kv3.1 glycoprotein. Electrophoretic migration of the various Kv3.1 proteins treated with PNGase F and neuraminidase verified that the glycosylation sites were occupied and that the *N*-glycans could be sialylated, respectively. The unglycosylated channel favored a different whole cell current pattern than the glycoform. Further the outward ionic currents of the unglycosylated channel had slower activation and deactivation rates than those of the glycosylated Kv3.1 channel. These kinetic parameters of the partially glycosylated Kv3.1 channels were also slowed. B35 cells expressing glycosylated Kv3.1 protein migrated faster than those expressing partially glycosylated and much faster than those expressing the unglycosylated Kv3.1 protein. These results have demonstrated that *N*-glycans of the Kv3.1 glycoprotein enhance outward ionic current kinetics, and neuronal migration. It is speculated that physiological changes which lead to a reduction in *N*-glycan attachment to proteins will alter the functions of the Kv3.1 channel.

## Introduction

Voltage-gated potassium channels (Kv) are essential components responsible for determining the intrinsic electrical excitability of neurons by repolarizing action potentials [1]. A less defined role of Kv channels is their non-conducting functions, such as cell-cell interactions, cell migration, and cell proliferation [2], [3]. The Kv3 channel subfamily members have two absolutely conserved *N*-glycosylation sites within the S1–S2 extracytoplasmic linker [4], [5]. The Kv3.1 channel, along with Kv3.3, have unique functional characteristics which include fast activation and deactivation rates, and channel activation at more depolarized potentials than other Kv channels [6]. These unique properties, as well as their expression in specific regions of the central nervous system, have implicated these Kv channels in the regulation of motor activity and refinement of motor tasks [7]. Further dysfunction and expression levels of these channels have been linked to diseases of the nervous system [8] and cancer [9], and therefore the modulation of conducting and non-conducting functions of the Kv3.1 channel is of considerable medical interest.

*N*-Glycans are attached to the Kv3 glycoproteins throughout the adult rat central nervous system [10]. Recently, it was demonstrated that the Kv3.1, 3.3, and 3.4, and Kv1.1, 1.2, and 1.4 proteins of adult rat brain, as well as Kv3.1 heterologously expressed in B35 cells, contain sialylated *N*-glycans [11]. To date, studies of *N*-glycosylation processing on Kv channels have been conducted utilizing non-excitable cell systems. Evidence showed that the absence of simple type *N*-glycans on Kv3.1 channels when expressed in *Spodoptera frugiperdae* (Sf9) insect cells causes slower activation rates relative to its glycosylated counterpart [5]. Additional studies have also shown that the *N*-glycan alters the gating function of Kv1.1 [12], [13], Kv1.2 [14], Kv10.1 channels [15], Shaker B K^+^ channel [16] and Kir1.1 [17], as well as influences proper trafficking of the Kv1.2 [14] and Kv1.4 channels [18].

Attachment of *N*-linked oligosaccharides to newly synthesized membrane proteins is the most ubiquitous protein co-translational modification in the lumen of the endoplasmic reticulum (ER) [19]. Further, the biological importance of the *N*-glycosylation pathway in mammalian physiology has been emphasized by the identification of congenital disorders of glycosylation in humans [20] and mutant glycosylation mice [21], [22], [23], [24]. It has also been suggested that ER stress, which would most likely alter *N*-glycosylation occupancy, may lead to several neurodegenerative diseases [25]. The human diseases and mouse models underscore the relevance of the *N*-glycosylation process in mammalian physiology.

The primary aim of this paper was to determine whether *N*-glycans attached to the Kv3.1 glycoprotein could impact the conducting and non-conducting roles of the Kv3.1 channel in neuronal-derived cells. For this purpose glycosylated (wild type), partially glycosylated (N220Q, N229Q) and unglycosylated (N220Q/N229Q) Kv3.1 proteins were expressed in rat B35 neuroblastoma cells. B35 cells represent a cultured neuronal cell model of the central nervous system [26]. Immunoband shift assays were used to characterize the *N*-glycans of various Kv3.1 proteins. Whole cell current recordings and wound healing assays were performed to examine outward ionic current kinetics and migration of Kv3.1 transfected B35 cells. We demonstrated that B35 cells expressing the unglycosylated Kv3.1 protein had outward ionic currents with slower activation and deactivation rates, and reduced cell migratory rates compared to those of the glycosylated Kv3.1 proteins, indicating that the *N*-glycans of the Kv3.1 glycoprotein influence conducting and non-conducting roles of the Kv3.1 channel.

## Results

### *N*-Glycans of the Kv3.1 protein were sialylated

**Figure 1:**
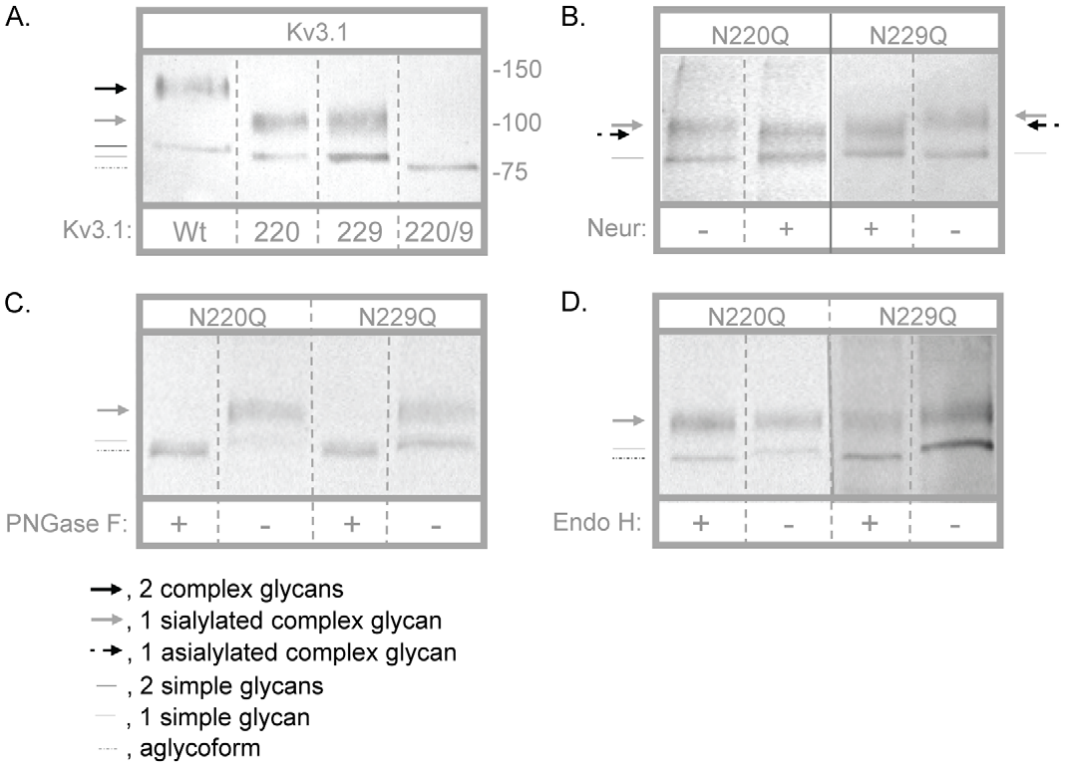
Characterization of Kv3.1 proteins expressed in B35 cells. Western blots of wild type (Wt), N220Q, N229Q, and N220Q/N229Q Kv3.1 proteins. Kv3.1 proteins were detected when heterologously expressed in B35 cells (A). Arrows and lines denote the type of *N*-glycan attached to the Kv3.1 protein. Assignments of the various glycosylated and unglycosylated Kv3.1 proteins were based on immunoband shifts produced by glycosidase treatment. N220Q and N229Q proteins were digested (+) and undigested (−) with neuraminidase (B), PNGase F (C) and Endo H (D). A solid line on image indicates that samples were run on a different blot (B). The numbers adjacent to the Western blots represent the Kaleidoscope markers (in KDa). Similar migration patterns were observed on at least three separate Western blots.

Recently, metabolic labeling studies showed that the Kv3.1 channel heterologously expressed in B35 cells generates the Kv3.1 glycoprotein with sialylated *N*-glycans [11]. Here we have utilized this same expression system to ascertain whether *N*-glycans associated with the Kv3.1 glycoprotein could influence conducting and non-conducting properties of the Kv3.1 channel. Wild type Kv3.1 protein has two *N*-glycosylation sites in the first extracellular loop which are occupied in native tissue and heterologous expression systems [4], [5], [10], [11]. Kv3.1 *N*-glycosylation mutants were constructed by highly conserved mutations of the Asn residues to Gln residues (N220Q/N229Q, N220Q and N229Q) [5]. Kv3.1 proteins tagged with the FLAG epitope were M2 immunoaffinity purified from stable transfected B35 cells. Partially purified Kv3.1 samples were then analyzed by Western blots (Figure 1). Wild type Kv3.1 migrated as a doublet with a predominant slower migrating band (≈132 kDa) and a very faint faster migrating band (≈88 kDa) (Figure 1A). As previously shown, the upper and lower bands represent attachment of two complex and simple *N*-glycans, respectively, to the Kv3.1 protein [11], [27]. When both sites were abolished (N220Q/N229Q), a single migrating species (≈81 kDa) was detected which corresponded to the unglycosylated Kv3.1 protein [5]. Two immunobands for each of the single *N*-glycosylation Kv3.1 mutants with similar electrophoretic mobility were detected (Figure 1A). The upper bands (≈106 kDa) (Figure 1A) were sensitive to neuraminidase (Figure 1B) and PNGase F (Figure 1C) and resistant to Endo H (Figure 1D). In contrast, the lower bands (≈84 kDa) (Figure 1A) were resistant to neuraminidase (Figure 1B) and sensitive to PNGase F (Figure 1C) and Endo H (Figure 1D). These results indicate that the upper band of the single mutants denote a sialylated complex *N*-glycan associated with the N220Q or N229Q Kv3.1 proteins while the lower band is a simple *N*-glycan. Further removal of sialic acid from the single mutants represents the attachment of one asialylated complex *N*-glycan to the N220Q or N229Q Kv3.1 proteins. To evaluate whether endogenous Kv3.1, 3.3 and 3.4 proteins could be detected in B35 cells, nontransfected B35 membrane proteins, as well as partially purified Kv3.1 protein samples, were probed with the various anti-Kv3 antibodies by Western blotting (not shown). In all cases, endogenous Kv3.1, 3.3, and 3.4 glycoproteins were not observed in nontransfected B35 cells nor were endogenous Kv3.3 and 3.4 proteins found to form heteromultimers with the heterologously expressed Kv3.1 glycoprotein. These results have shown that both sites could be occupied by sialylated *N*-glycans, and that the occupancy of these sites was independent of one another. The removal of one or even both of these sites was also not observed to dramatically alter Kv3.1 protein expression, similar to that described in Sf9 cells [5]. Based on the most plausible explanations of the Western blotting studies, wild type, N220Q, N229Q, and N220Q/N229Q Kv3.1 proteins will be referred to as glycosylated, partially glycosylated, and unglycosylated Kv3.1 channels, respectively, throughout the text.

### Comparison of whole cell currents of glycosylated and unglycosylated Kv3.1 channels

**Figure 2:**
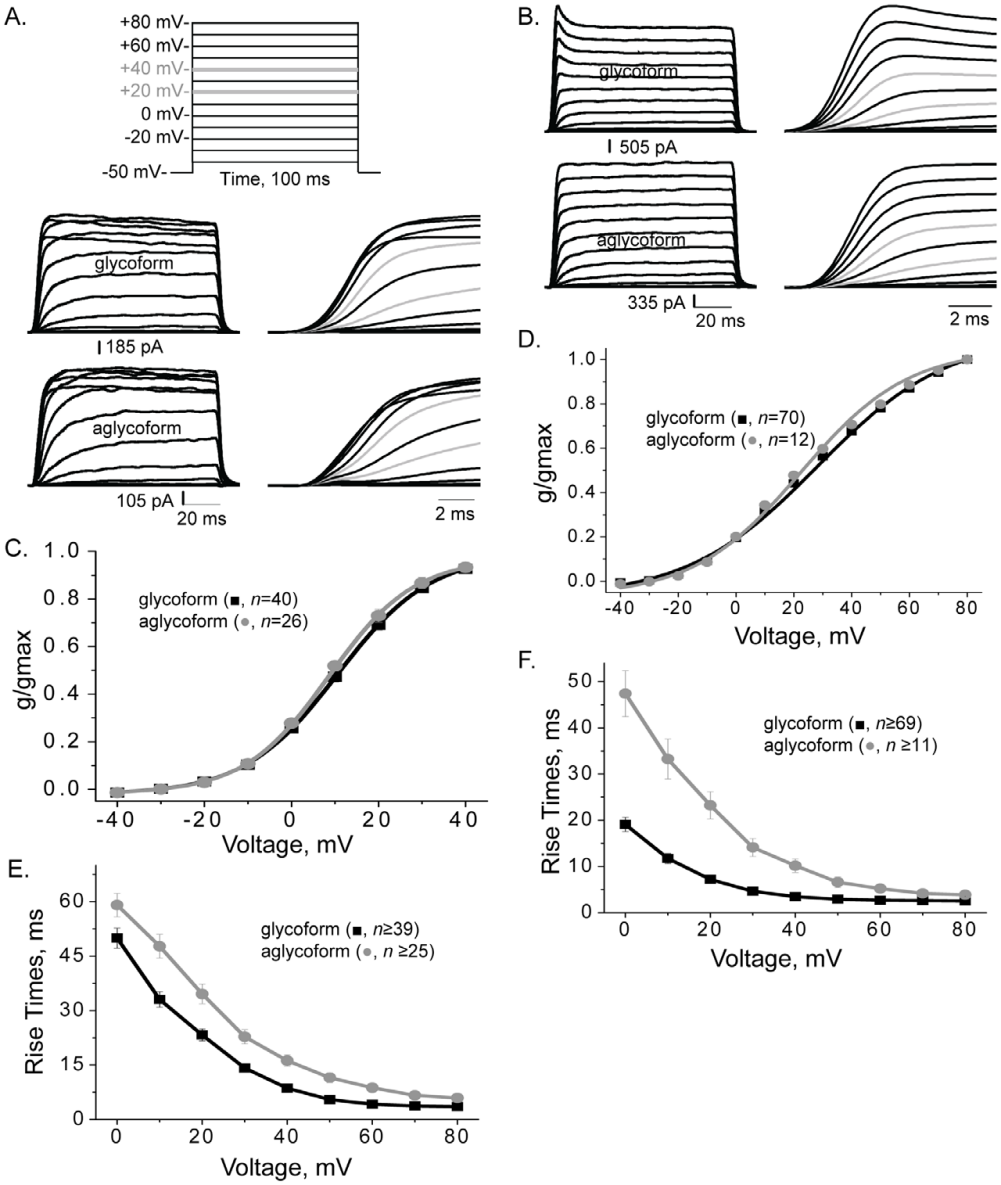
Aglycoform displayed slower activation kinetics of ionic currents than glycoform. Whole cell currents for glycosylated (A, middle panel; B, top panel) and unglycosylated (A and B, bottom panels) Kv3.1 proteins were elicited from the indicated voltage protocol (A, top panel). Whole cell currents were scaled for inactivating (A) and non-inactivating (B) current types from B35 cells expressing glycosylated and unglycosylated Kv3.1 proteins. Right panels show traces at expanded time scales and grey lines denote currents at +40 and +60 mV. Traces were scaled to show differences in activation kinetics. Conductance-voltage (g/gmax) curves of both inactivating (C) and non-inactivating (D) current types for glycosylated and unglycosylated Kv3.1 channels. Rise times of inactivating (E) and non-inactivating (F) currents types. *n* represents number of cells.

An activation protocol with 100 ms voltage steps from −40 to +80 mV in 10 mV increments and a resting membrane potential of −50 mV was employed to determine the voltage dependence of activation, rise times, and activation time constants (Figure 2A, top panel). Two types of whole cell currents have been reported for the Kv3.1 channel expressed in oocytes [28], [29], [30], [31], mammalian cells [6], [32], [33], [34]and infected Sf9 cells [5]. The inactivating current type represents saturation of current amplitudes at more depolarized potentials while the noninactivating current type lacks saturation. Both inactivating (Figure 2A, middle and bottom panels) and noninactivating (Figure 2B) current types were observed for B35 cells heterologously expressing glycosylated (middle and top panels) and unglycosylated (bottom panels) Kv3.1 proteins. In both cases, whole cell current recordings were scaled to illustrate differences in activation kinetics. The predominant current type for glycosylated Kv3.1 was the noninactivating current while that for unglycosylated Kv3.1 was the inactivating current. Further the noninactivating currents of glycosylated Kv3.1 channels usually had a transient current peak at the initial phase of the sweep (Figure 2B, top panel) while the unglycosylated channel usually lacked the transient peak (Figure 2B, bottom panel).

The conductance-voltage (G–V) relationship were fitted with the Boltzmann isotherm for inactivating (Figure 2C) and noninactivating (Figure 2D) current types. In all cases, currents were detected when the applied test potential was greater than −30 mV. However, current amplitudes reached saturation much more readily for inactivating current patterns than noninactivating current patterns. The membrane conductance determined from the peak current amplitude versus applied test potential indicated that a similar depolarization was required for 50% of glycosylated and unglycosylated Kv3.1 channels to reach activation (Table 1). It also appeared that a comparable amount of channels were activated as the applied voltage was increased for glycosylated and unglycosylated Kv3.1 channels.

**Table 1:** Electrophysiological parameters of glycosylated, unglycosylated, and partially glycosylated forms of the Kv3.1 channel in B35 cells.

Parameter	Wild type Kv3.1	N220Q/N229Q	N220Q	N229Q
**V_0.5_ (mV)**				
InactivatingNoninactivating	10.1±0.7 (*n* = 40)28.1±0.7 (*n* = 70)	8.1±0.6 (*n* = 26)22.3±0.4 (*n* = 12)	9.2±0.8 (*n* = 17)30.7±0.6 (*n* = 9)	13.1±0.8 (*n* = 12)27.5±0.5 (*n* = 29)
**dV (mV)**				
InactivatingNoninactivating	10.5±0.5 (*n* = 40)22.6±0.4 (*n* = 70)	9.6±0.3 (*n* = 26)18.7±0.5 (*n* = 12)	9.9±0.4 (*n* = 17)19.8±0.6 (*n* = 9)	10.8±0.7 (*n* = 12)24.3±0.6 (*n* = 29)
**RT (ms) at +40 mV**				
InactivatingNoninactivating	8.6±0.5 (*n* = 40)3.5±0.2 (*n* = 70)	15.9±1.5 (*n* = 26)*10.2±1.5 (*n* = 12)*	10.6±0.9 (*n* = 17)*3.2±0.4 (*n* = 9)	12.7±1.6 (*n* = 12)*3.1±0.1 (*n* = 29)
**τ_on_ (ms) at +40 mV**				
InactivatingNoninactivating	4.0±0.3 (*n* = 40)1.6±0.2 (*n* = 70)	7.5±0.8 (*n* = 26)*7.4±1.2 (*n* = 12)*	5.3±1.0 (*n* = 17)1.4±0.4 (*n* = 9)	6.6±0.9 (*n* = 12)*1.2±0.1 (*n* = 29)
**τ_off_ (ms) at +40 mV**				
InactivatingNoninactivating	4.1±0.2 (*n* = 28)3.6±0.1 (*n* = 54)	6.0±0.3 (*n* = 19)*5.6±0.2 (*n* = 9)*	4.7±0.4 (*n* = 9)4.0±0.3 (*n* = 8)	5.1±0.2 (*n* = 9)*3.9±0.2 (*n* = 26)
**% current remaining**				
at +40 mV	51.0±2.3 (*n* = 18)	62.3±3.1 (*n* = 10)*	60.2±2.7 (*n* = 14)*	64.8±4.1 (*n* = 10)*
**% current type**				
Inactivating	36%	68%	65%	29%
Noninactivating				
w/transient peaks	42%	8%	27%	54%
w/o transient peaks	22%	24%	8%	17%

The biophysical parameters are shown for the inactivating current and noninactivating current types. Data are presented as the mean ± S.E. and *n* represents the number of cells expressing Kv3.1 currents. V_0.5,_ voltage at which 50% of activation is reached based on conductance-voltage curve determined from the current amplitude of the peak current; dV, slope factor based on conductance-voltage curve; RT stands for rise times, τ_on_, activation time constant; τ_off_, deactivation time constant; % current remaining corresponds to the percent of current remaining near the last 10 ms of the sweep relative to the peak current amplitude for inactivating current types; % current type represents a given type of current divided by total current types.

A value of P<0.05 was considered significant (*).

### Glycosylated and unglycosylated Kv3.1 have different activation kinetics of ionic currents

To illustrate differences in activation kinetics for glycosylated and unglycosylated Kv3.1 channels, the time scales were expanded for inactivating (Figure 2A, middle and bottom right panels) and noninactivating (2B, right panels) current types. The rise times were slowest at the lowest applied test potential and fastest at the highest potential for both Kv3.1 forms which indicates the voltage dependence of the channels. Rise times were faster for the glycosylated Kv3.1 channel than the unglycosylated Kv3.1 channel at and beyond 0 mV for both inactivating (Figure 2E) and noninactivating (Figure 2F) current types. Further the activation time constants at +40 mV for both current types of the glycosylated Kv3.1 channel were faster than those of the unglycosylated Kv3.1 channel (Table 1). These results showed that the activation rates of the outward ionic currents of the Kv3.1 channel were faster for the glycoform than the aglycoform.

### Glycosylated and unglycosylated Kv3.1 have different deactivation kinetics of ionic currents

**Figure 3:**
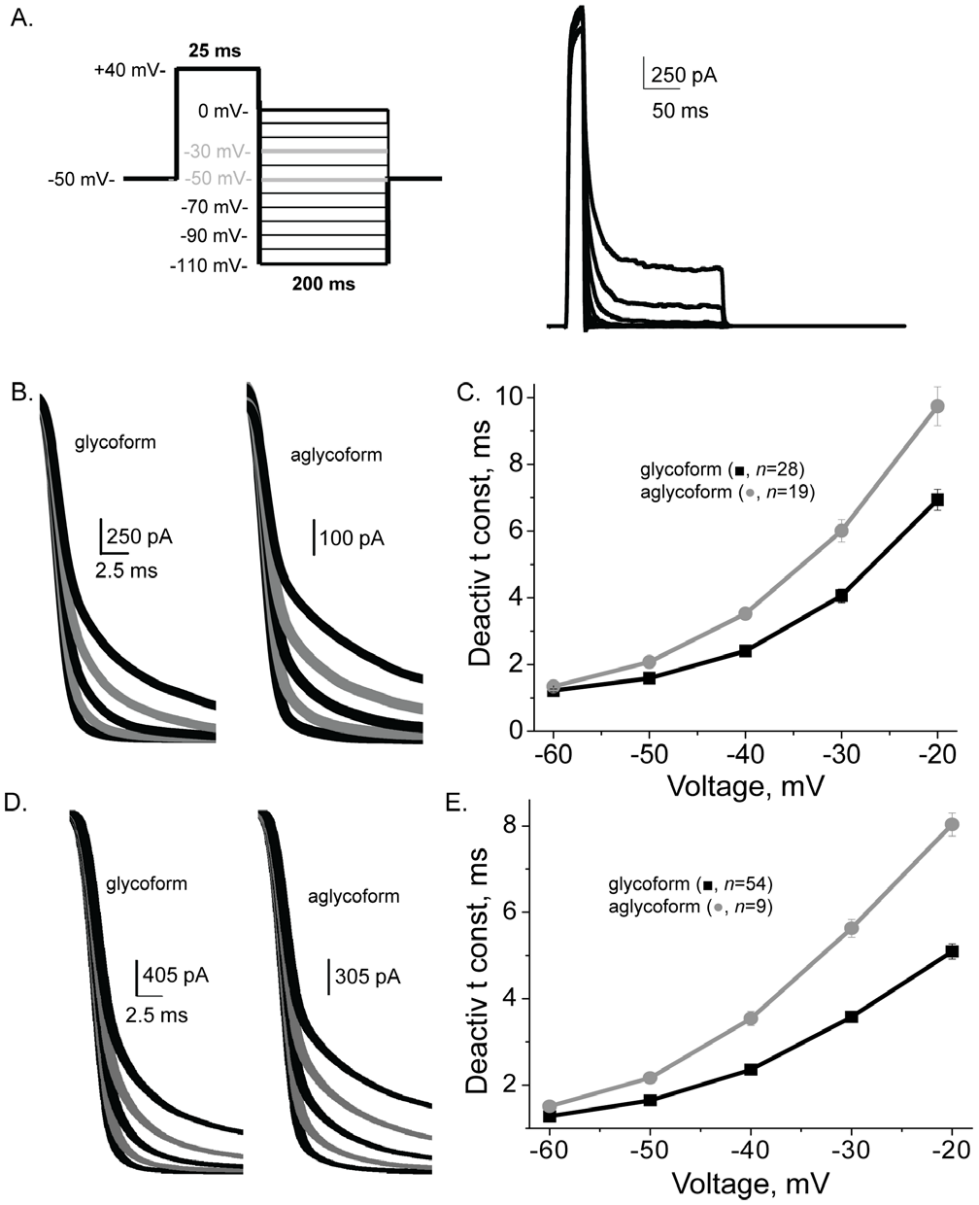
Deactivation rates of ionic currents were slower for aglycoform than glycoform. A deactivation voltage protocol (A, left panel) was utilized to obtain deactivation currents for B35 cells expressing the glycosylated (A, right panel) and unglycosylated. Scaled deactivation currents from transfected B35 cells expressing either inactivating (B) or non-inactivating (D) current types. Grey lines denote currents at −30 and −50 mV. Traces were scaled to show differences in deactivation kinetics. Deactivation time constant vs. voltage plot of B35 cells expressing glycosylated and unglycosylated Kv3.1 channels for inactivating (C) and non-inactivating (E) currents types.

The deactivation rates of Kv3 channels are rapid [6]. To examine the deactivation kinetics of the glycosylated and unglycosylated Kv3.1 channels, transfected B35 cells were clamped at +40 mV for 25 ms to activate Kv3.1 channels, and subsequently the channels were deactivated by clamping the cells to less depolarized potentials (Figure 3A). Both glycosylated and unglycosylated forms of the Kv3.1 channel displayed rapid deactivation rates for inactivating (Figure 3B and 3C) and noninactivating (Figure 3D and 3E) current types. However, the deactivation rates of the glycosylated Kv3.1 channel were significantly faster than those of the unglycosylated Kv3.1 channel for both current types. These results demonstrated that *N*-glycans of the Kv3.1 glycoprotein enhance deactivation rates of the outward ionic currents.

### Ionic currents of the glycoform inactivated to a greater extent than aglycoform

**Figure 4:**
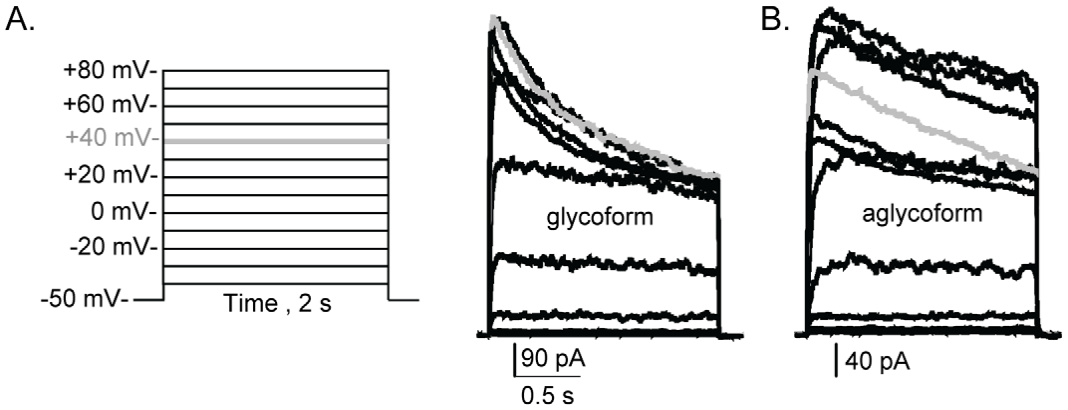
Ionic currents of the aglycoform inactivated to a lesser extent than glycoform. Whole cell currents were elicited from the shown voltage protocol (A, left panel) for B35 cells expressing glycosylated (A, right panel) and unglycosylated (B) Kv3.1 proteins. Traces were scaled to show differences in inactivation kinetics. Grey lines denote currents at +40 mV.

An activation voltage protocol (Figure 4A, left panel) with pulse duration of 2 s at various voltage steps was utilized to examine the inactivation kinetics of inactivating current types for glycosylated (Figure 4A, right panel) and unglycosylated (Figure 4B) Kv3.1 channels. The whole cell recordings showed that initially the currents quickly increased with time, and then gradually decreased as the duration of the pulse continued. Additionally, the decrease in current amplitudes at the end of pulses relative to the maximum current amplitudes was greater for the glycosylated Kv3.1 channel than unglycosylated. For example, the percent of current remaining for glycosylated Kv3.1 was about 51% when the test potential was +40 mV while that of unglycosylated Kv3.1 was about 62% (Table 1). These results indicated that *N*-linked carbohydrates influence inactivation rates of the outward ionic currents produced by the Kv3.1 channel.

### Cumulative inactivation was slightly greater for unglycosylated Kv3.1 than glycosylated Kv3.1

**Figure 5:**
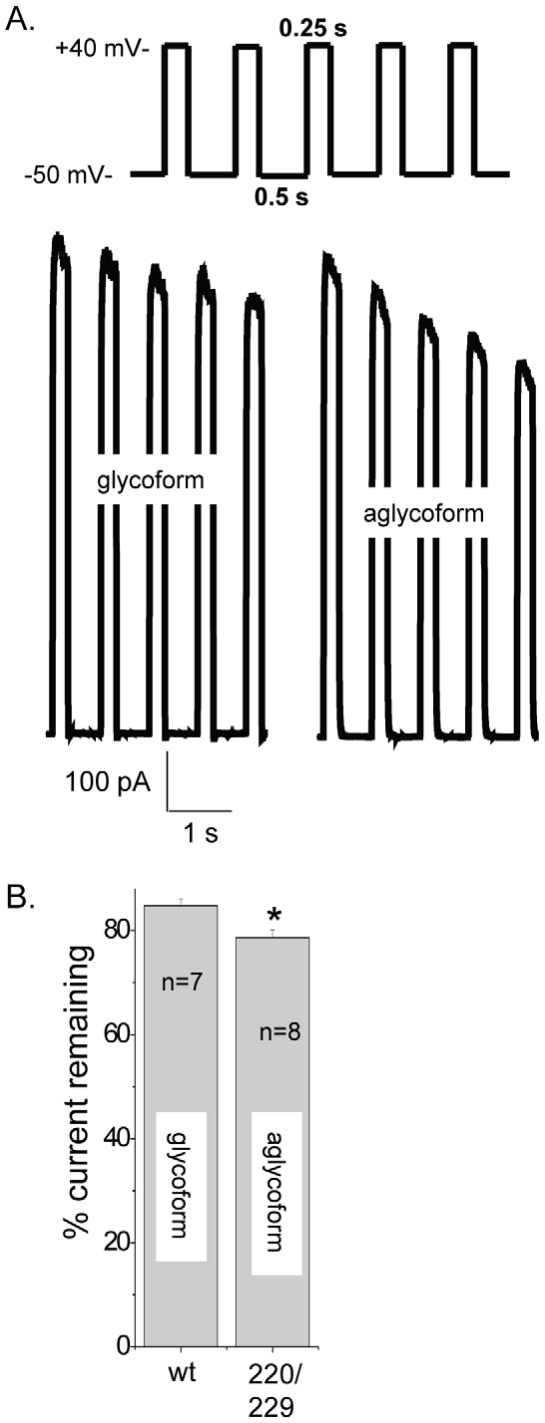
Ionic currents of aglycoform decreased more readily during repetitive depolarization pulses than glycoform. Currents were elicited by a train of five depolarizing voltage steps to +40 mV once every 525 ms, from a holding potential of −50 mV (A, top panel) for B35 cells expressing glycosylated (A, bottom-left panel) and unglycosylated (A, bottom-right panel) Kv3.1 channels. A bar graph representing the percent of peak current amplitude remaining after the fifth pulse relative to peak current amplitude of initial pulse for the various Kv3.1 channels (B). Asterisks indicate significant differences in mean values at a probability of *P*<0.01 from that of glycosylated Kv3.1.

The whole cell current of the glycosylated Kv3.1 channel lacks sensitivity to repetitive depolarization pulses [35], [36]. Voltage dependent currents were elicited by a train of five depolarizing voltage steps to +40 mV once every 500 ms, from a holding potential of −50 mV for glycosylated (Figure 5A, bottom left panel) and unglycosylated (Figure 5A, bottom right panel) Kv3.1 channels. A bar graph of the percent of peak current remaining after the fifth repetitive depolarization pulse relative to the first pulse reveals that the unglycosylated Kv3.1 channel is more sensitive to repetitive activation than the glycosylated Kv3.1 channel (Figure 5B).

### Analysis of the partially glycosylated Kv3.1 channels

Similar to the glycosylated and unglycosylated Kv3.1 channels, the partially glycosylated Kv3.1 channels expressed both types of whole cell current. Inactivating currents were more prevalent than noninactivating currents for the N220Q channel, like the unglycosylated channel. Alternatively, the major current type expressed by the N229Q channel was the noninactivating current, similar to the glycosylated Kv3.1 channel. For both partially glycosylated Kv3.1 forms most of the noninactivating currents had transient peaks, like the glycosylated Kv3.1 channel.

The number of channels activated as the applied voltage was increased for the partially glycosylated Kv3.1 channels were similar to the glycosylated Kv3.1 channel (Table 1). Further the amount of depolarization required for 50% of the partially glycosylated Kv3.1 channels to reach activation was like the glycosylated Kv3.1 channel. In contrast, the inactivating currents of the partially glycosylated Kv3.1 channels had different activation and deactivation rates than glycosylated Kv3.1 while those of noninactivating currents were similar (Table 1). Both of the partially glycosylated Kv3.1 channels displayed significantly slower decreases in outward current with time compared to the glycosylated Kv3.1 channel for the inactivating current type (Table 1). These results demonstrated that the partially glycosylated Kv3.1 channels share some biophysical properties with the glycosylated Kv3.1 channel and others with the unglycosylated Kv3.1 channel.

### Glycosylated Kv3.1 enhances neuronal migratory rates

**Figure 6:**
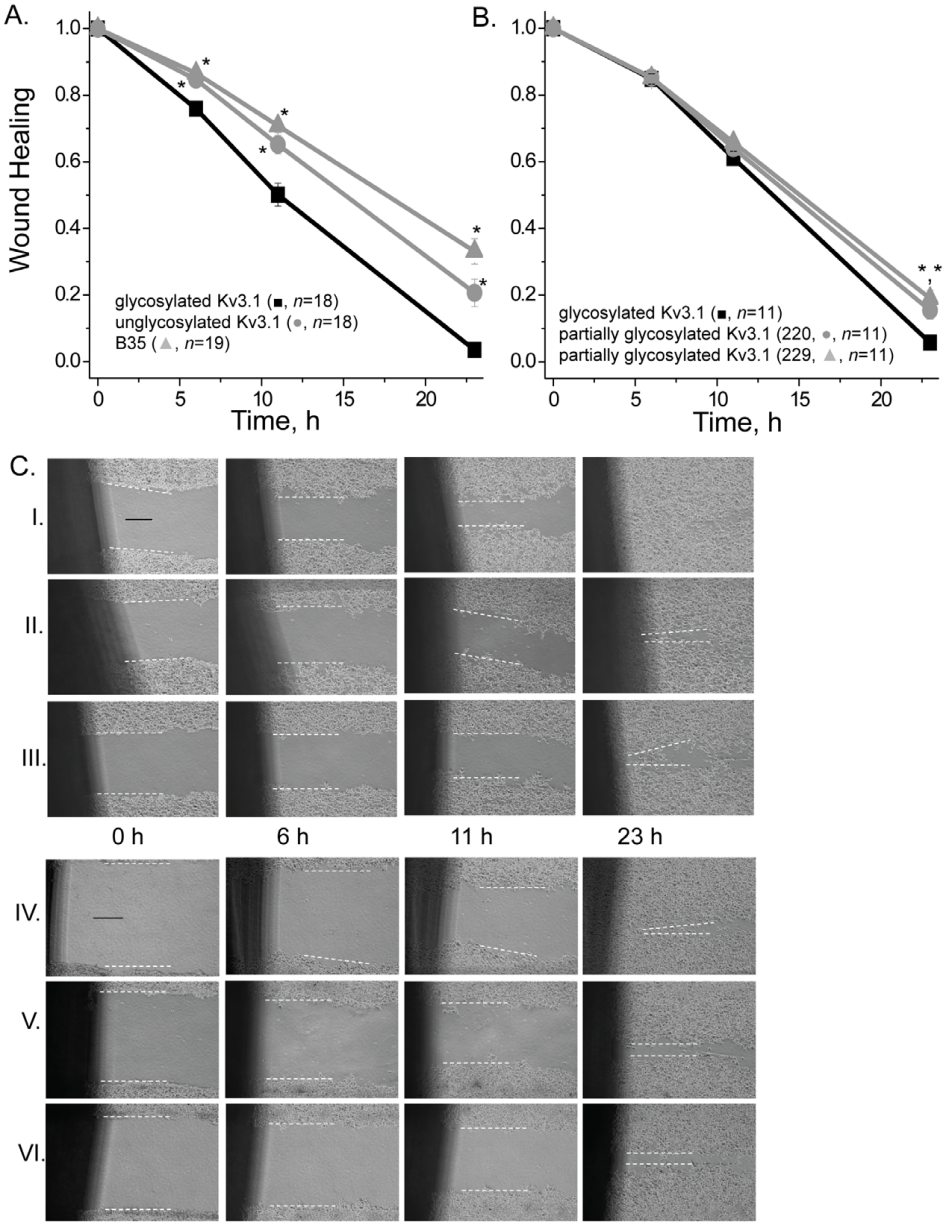
Kv3.1 protein associated with *N*-glycans enhances neuronal cell migration. Wound width was determined and then normalized at 0, 6, 11 and 23 h for glycosylated and unglycosylated Kv3.1 transfected and non-transfected B35 cells (A). Similar experiments were also performed for glycosylated, and partially glycosylated Kv3.1 glycoproteins (N220Q, and N229Q) transfected B35 cells (B). Data were expressed as the mean +/− SEM. Asterisks indicate significant differences in mean values at a probability of *P*<0.01 from that of glycosylated Kv3.1. Images were obtained at 0 h, 6 h, 11 h and 23 h of the generated wound for group I: wild type Kv3.1 (row I), N220Q/N229Q (row II) transfected B35 cells and non-transfected B35 cells (row III); and group II: wild type Kv3.1 (row IV), N220Q (row V) and N229Q (row VI) (C). The distance between the two white dashed lines represents wound width for each image. This distance becomes smaller as time increases, representing the rate of cell migration. *n* represents number of cell wounds. The experiments were conducted on three separate occasions. The solid black line represents a 25 µM scale bar.

Cell wound healing assays were performed for two distinct experimental groups (Figure 6). In both cases, the various B35 cell models were compared to the B35 cell model expressing wild type Kv3.1 protein. Group I represents B35 cells transfected with recombinant mammalian vectors encoding wild type or N220Q/N229Q Kv3.1 cDNAs, as well as non-transfected B35 cells (Figure 6A and Figure 6C, Rows I, II, III). Group II are B35 cells expressing wild type Kv3.1, N220Q, or N229Q proteins (Figure 6B and Figure 6C, Rows IV, V, VI). Wound width (in µM) was determined at 0, 6, 11, and 23 hours. Although mean values of initial wounds for each group were quite similar (Group I: wt Kv3.1, 65±3 µM; N220Q/N229Q, 64±2 µM; B35, 67±2 µM; Figure 6C, Row I, II, III; Group II: wt Kv3.1, 91±2 µM; N220Q, 90±2 µM; N229Q, 93±2 µM; Figure 6C, Row IV, V, VI), the wound width was normalized so initial time points would be identical. The wound healing versus time curves demonstrate that B35 cells expressing glycosylated Kv3.1 have faster migratory rates relative to those expressing unglycosylated Kv3.1, and non-transfected B35 cells, as well as the partially glycosylated Kv3.1 proteins. Additionally, it appears that unglycosylated Kv3.1 expressing cells migrate slightly faster than non-transfected B35 cells. Taken together, these results indicate that the *N*-glycans attached to the Kv3.1 protein alter neuroblastoma cell migration.

## Discussion

In this study, we have demonstrated that *N*-glycans of the Kv3.1 glycoprotein influence conducting and non-conducting functions of the Kv3.1 channel. Heterologous expression of wild type, N220Q, N229Q, and N220Q/N229Q Kv3.1 proteins in B35 cells were utilized to generate Kv3.1 proteins with and without *N*-glycans. Previously, we have shown that sialylated *N*-glycans associated with the Kv3.1 glycoprotein in transfected B35 cells and adult rat brain [11]. Immunoband shift assays of wild type and mutant Kv3.1 proteins revealed that both sites of the Kv3.1 protein expressed in B35 cells could be occupied by sialylated *N*-glycans. Therefore, transfected B35 cells are a feasible neuronal-derived cell expression system to evaluate the role of the Kv3.1 protein with two, one, and zero *N*-glycans on conducting and non-conducting functions of the Kv3.1 channel.

To date, the role of *N*-glycans associated with the Kv3.1 glycoprotein on favoring a whole cell current pattern has not been established. Noninactivating currents with transient peaks were originally identified when Kv3.1 was heterologously expressed in HEK cells [32], and later in other mammalian cells [33], [34]. Noninactivating currents without transient peaks have also been well documented [6], [33], [34]. Our results showed that noninactivating currents with and without transient peaks were expressed by the various Kv3.1 channels. However, the noninactivating currents of glycosylated (66%), and partially glycosylated (N220Q, 78%; N229Q, 76%) Kv3.1 channels had transient peaks the majority of the time while those for the aglycoform (25%) was much less (Table 1). Furthermore, this type of current was detected for glycosylated Kv3.1 (42%), partially glycosylated (N229Q, 54%; N220Q, 27%), and much lower for the aglycoform (10%) when including all current types. Thus, these results argue that the attachment of at least one *N*-glycan to the Kv3.1 protein greatly enhances the production of both types of noninactivating current, and furthermore those with transient peaks.

Biophysical properties of heterologously expressed Kv3.1 protein are utilized to identify Kv3.1 channel current in native tissue. This report has established biophysical properties of Kv3.1 channels with and without *N*-linked oligosaccharides in B35 cells. Previously, it was reported that the wild type Kv3.1 channel heterologously expressed in various cell lines had a range of values for V_0.5_ from 10 mV to 18 mV, and dV from 8 mV to 11 mV [5], [6], [37] which are quite similar to those determined for the inactivating current type of wild type and glycosylation mutant Kv3.1 channels expressed in B35 cells. On the contrary, the range of values for V_0.5_ from 22 mV to 31 mV, and dV from 19 mV to 24 mV for the noninactivating current type of wild type and glycosylation mutant Kv3.1 channels were higher. The higher values reflect the lack of saturation of current amplitudes at more depolarized potentials since the threshold potential of the various Kv3.1 channels were similar. Overall, the ionic current kinetic parameters for the glycosylated Kv3.1 channel also compared more to those reported for other mammalian cells [6], [34], [37] than the aglycoform. It was observed that the Kv3.1 protein deficient in sialylated *N*-glycans had slower activation and deactivation rates, and faster inactivation kinetics compared to glycosylated Kv3.1 channels. Further the slowed deactivation kinetics and enhanced inactivation kinetics of the aglycoform was reflected by the reduction in current during repetitive depolarization for unglycosylated Kv3.1 channel. Therefore, this study establishes biophysical parameters for Kv3.1 proteins with two, one, and zero *N*-glycans in neuronal-derived cells.

Outward ionic current kinetics of the Kv3.1 channel depended on the number of *N*-glycosylation sites occupied. The deactivation and activation rates were slowed when the Kv3.1 protein lacked both *N*-glycans. This delay in the opening and closing of Kv3.1 channels with one *N*-glycan was also observed for the inactivating current type but was less dramatic. Moreover, shifts in the activation and deactivation kinetics were not due to shifts in the voltage midpoints of channel activation. These results argue that the gating stabilizing theory, not surface potential theory, is more accurate in describing the gating mechanism [1], [13] of the Kv3.1 channel. Previous findings of the Kv1.1 [12], [13], Kv1.2 [14] and Drosophila ShB [16] channels indicated that the *N*-glycans modulated these channels by the surface potential theory, along with the gating stabilizing theory. An earlier study of the Kir1.1 channel also showed that the open state was destabilized by the absence of *N*-glycosylation [17]. Taken together, these results indicated that the conducting properties of the Kv3.1 channel can be altered by the amount of occupied *N*-glycosylation sites of the Kv3.1 protein.

A non-conducting function of Kv channels is cell migration [2], [3], [7], [38] which is a critical process in the development and maintenance of multicellular organisms. The Kv3.1 channel was shown to play a major role in regulation of oligodendrocyte progenitor cell development and axon myelination [38]. Further specific block of Kv3.1 or Kv3.1 knock-out mice inhibited proliferation and migration of oligodendrocyte progenitor cells. Additionally, diseased Kv3.3 mutations have been linked to development disorders to adult-onset neurodegeneration, suggesting Kv3 proteins as candidates for neurodegenerative diseases [8]. In this study, it was shown that migratory rates of the neuroblastoma cells were altered by the absence of 1 or 2 *N*-glycans of the Kv3.1 protein relative to that with both *N*-glycans. Previous studies have shown that cell migration is regulated by cell adhesion and changes in transmembrane flow and that the integrin/channel complex contributes to this role. Since *N*-glycans participate in cellular adhesion [39] it may be that glycosylated ion channels regulate cell migration independently or along with the integrin receptor. These results demonstrate that the *N*-glycans associated with the Kv3.1 protein modify neuroblastoma cell migration.

Here we show that *N*-linked glycans of the Kv3.1 glycoprotein have an impact on conducting and non-conducting properties of the Kv3.1 channel in B35 cells. Our results are novel in several respects. Firstly, opening and closing rates of the Kv3.1 channel were faster when both *N*-glycosylation sites of the Kv3.1 protein were occupied. Lastly, the number of *N*-glycans associated with the Kv3.1 protein influenced neuroblastoma cell migratory rates. We speculate that the expression of specific Kv3.1 glycoforms may influence action potential waveforms, and furthermore that some patients suffering from congenital disorders of glycosylation may have altered conducting and non-conducting functions of the Kv3.1 channel.

## Materials and Methods

### Molecular Biology

Rat Kv3.1 recombinant pcDNA3.1 mammalian expression vectors (3′FLAG-Kv3.1-pCDNA3.1, 3′FLAG-N220Q/N229Q-pCDNA3.1, 3′FLAG-N220Q-pCDNA3.1, and 3′FLAG-N229Q-pCDNA3.1) were constructed for heterologous expression in B35 neuroblastoma cells. The highly conserved mutations generate *N*-glycosylation Kv3.1 mutants in which either one or both native *N*-glycosylation sites at positions 220–222 (NKT) and 229–231 (NGT) were abolished. The FLAG epitope tagged Kv3.1 cDNA molecules were acquired from previous study utilizing Sf9 cells [5]. In brief, each of the respective Kv3.1 molecules were removed from their respective Baculovirus expression vectors by EcoRI (New England BioLabs, Ipswich, MA, USA) digestion and subcloned into EcoRI digested pCDNA3.1 vector. In all cases, orientation of Kv3.1 cDNA molecules was determined with SmaI digests (New England BioLabs, Ipswich, MA, USA). Standard procedures were followed for DNA amplification, DNA isolation, and subcloning [40].

### B35 cell culture and the establishment of stable cell lines

B35 neuroblastoma cells (rat central nervous system, derived) were obtained from American Type Culture Collection (Manassas, VA, USA) and maintained as previously described [11]. For the production of stable cell lines expressing the various forms of the Kv3.1 protein, B35 cells of >75% confluency were transfected with neomycin selectable pCDNA3.1 expression plasmids encoding wild type Kv3.1, N220Q/N229Q, N220Q or N229Q using Lipofectamine™ 2000 (Invitrogen, Carlsbad, CA, USA), similar to our previous study for the wild type Kv3.1 protein [11]. For whole cell measurements, stable transfected B35 cells were seeded onto small glass chips (Fisher Scientific, Suwanee, GA, USA) in a 35 mm dish (Fisher Scientific, Suwanee, GA, USA) for at least 15 h.

### M2 immunoaffinity purified Kv3.1 and glycosidase digestion reactions

To evaluate occupancy of the *N*-glycosylation sites and the type of *N*-glycan of Kv3.1 proteins, the various Kv3.1 proteins from stable transfected B35 cells were immunoaffinity purified as previously described [11]. Glycosidase digestion reactions were performed as described [11]. Neuraminidase (0.83 U/µL) (New England Biolabs, Ipswich, MA, USA), Endo H (50 U/µL) (New England Biolabs, Ipswich, MA, USA) and PNGase F (20 U/µL) (New England Biolabs, Ipswich, MA, USA) were used for digestions. For control reactions buffer was substituted for the enzyme.

### Immunoblotting

M2 immunoaffinity purified samples of Kv3.1 proteins in reducing SDS sample buffer (2X) were subjected to electrophoresis for 90–110 min at 20 mAmps on 10% SDS gels. Electrophoresed proteins were transferred to Immobilon-P PVDF membranes (Millipore, Billercia, MA, USA) at 175 mAmps for 90–240 min. Blots were then incubated at room temperature for 20 min in blocking buffer (PBS, 3% BSA with 0.1% Tween 20) followed by incubation for 2 h with polyclonal rabbit anti-Kv3.1, anti-Kv3.3, or anti-Kv3.4 antibodies (Alamone Labs, Jerusalem, Israel) or overnight with mouse anti-Kv3.1 antibody (NeuroMab). The specificity of the anti-Kv3.1 antibody was previously described [5], [10], [11], [27]. Subsequently blots were washed, and then incubated overnight at 4°C with their specific alkaline phosphate conjugated secondary antibody. Finally, immunobands were developed with ImmunO alkaline phosphatase substrate (MP Biomedicals, Irvine, CA, USA).

### Wound Healing Assays

B35 neuroblastoma cells non-transfected and transfected with wild type Kv3.1, N220Q/N229Q, N220Q, and N229Q were seeded in equal concentrations onto 60 mm CellBind culture dishes (Corning, Corning, NY, USA). After about 65 hrs complete medium was removed from the dishes and cell wounds were made in the monolayer using a beveled 200 µl pipet tip (Fisher Scientific, Suwanee, GA, USA). Cells were rinsed twice with DMEM and replaced with 3 mL of complete medium. Images of the cells were obtained at various time points from 0 to 38 hours on an Olympus IX 50 microscope using a 10X objective. Wound width (in µM) was determined at 0, 6, 11, and 23 hours. The wound width measurement involved determining the distant between two 60 µM lines which were placed at opposite sides of the wound, as indicated by the white dotted lines in Figure 6C, for each image. In some cases, images at 40X between 11 and 15 hours were acquired to monitor changes in morphology at the leading and trailing edge of the cells. In all cases, some cells could be observed to have a pyramidal shape (not shown), as expected for migrating cells.

### Whole cell recordings

Electrophysiological measurements were obtained from stable transfected B35 cells using the whole cell configuration of the patch clamp technique. Whole cell K^+^ currents were recorded at room temperature with an Axopatch 200B amplifier (Axon Instruments, Sunnyvale, CA, USA) controlled by CLAMPEX 9.0 software (Axon Instruments, Sunnyvale, CA, USA) running on a Dell Optiplex GX270 Pentium desktop computer using a Digidata 1322A analog-to-digital interface (Axon Instruments, Sunnyvale, CA, USA) [5]. Whole cell recordings were primarily conducted in pairs to reduce possible differences due to passage number and cell treatment. These differences were minimized by conducting the cell transfections and whole cell recordings in parallel for each pair. The two pairs were wild type and N220Q/N229Q Kv3.1 channels, and the two single mutant channels. In some cases, whole cell recordings of either wild type Kv3.1 or N220Q/N229Q were conducted alongside the single mutants. The external bath solution utilized during recordings had the following composition (in mM): 5 potassium aspartate, 135 sodium aspartate, 1 MgCl_2_ hexahydrate, 10 Mes, 60 mannitol (pH 6.3). The measured osmolarity of the external bath solution was 295–312 mOsm. Borosilicate glass tubing (Sutter Instruments, Novato, CA, USA) was pulled with a P-97 Flaming-Brown micropipette puller (Sutter Instruments, Novato, CA, USA) and fire polished with a MF-830 microforge (Narishige, McHenry, IL, USA) to obtain patch pipettes with a resistance of 4–9 MΩ when filled with intracellular solution. The intracellular solution contained (in mM): 140 potassium aspartate, 10 EGTA, 5 MgCl_2_ hexahydrate, 10 HEPES, 50 mannitol (pH 7.2). The measured osmolarity of the internal pipette solution was 320–340 mOsm similar to those previously used [5]. Both cell capacitance and series resistance was compensated for throughout the course of experiments. The patch pipette silver/silver chloride wire was connected to the input of an Axopatch 200B amplifier through a salt bridge (plastic pipette tip filled with 1% Agarose in 3.0 M KCl). All Kv3.1 currents were sampled at 10 kHz subsequent filtering at 1 kHz. Whole cell current recordings were accepted during those experiments only if membrane seal resistance was ≥1GΩ, maximum current amplitudes were ≥400 pA, and minimal cell capacitance was displayed after compensation. The endogenous currents (about 3.3 pA/pF) were minor in nontransfected B35 cells (the current amplitude was 37.9±4.2 pA with a mean capacitance of 11.6±1.6 pF, *n* = 21) compared to the Kv3.1 transfected B35 cells which had current densities ranging from 88–163 pA/pF and 239–401 pA/pF for inactivating and noninactivating current types, respectively. Therefore, the conductance-voltage relationships and kinetics of the outward ionic currents from transfected cells were attributed to the Kv3.1 channel. Whole cell recordings were acquired from several voltage clamp protocols as described in the text of the result section.

### Data analysis

Digitized whole cell current recordings from B35 cells were analyzed using the CLAMPFIT 9.0 analysis program (Axon Instruments, Sunnyvale, CA, USA) [5]. Normalized conductance-voltage relationships were fitted with a Boltzmann equation of the form: G  =  G_max_/[1 + exp(V_0.5_ - V_m_)/q] where q represents the slope factor, V_m_ stands for the test potential, V_0.5_ is the potential at which the conductance was half maximal, G is the conductance and G_max_ is the maximal conductance. For activation kinetics, whole cell current recordings were analyzed by both rise times and activation time constants. Rise times represent the time required for the current to rise from 10% to 90% of its peak current at a given applied test potential. Activation time constants were fitted with a single exponential function at the various test potentials. The mean deactivation time constant (tau) was determined by fitting the current at the applied test potentials with a single exponential. For inactivation kinetics, the percentage of current remaining at +40 mV was determined by dividing the peak current amplitude by the current remaining near the last 10 ms of the +40 mV sweep. For use dependence of the channel, the percent of current remaining was the peak current amplitude of the initial pulse divided by that of the fifth pulse.

Whole cell current and wound healing graphs were generated using Origin 7.5 (OriginLab Corporation, Northampton, MA, USA). Data are presented as the mean ± S.E. and *n* represents the number of cells tested or number of cell wounds. Student's t-test was utilized to evaluate the statistical comparisons. One-way ANOVA was used to evaluate statistical significance when more than two groups were compared. Statistical significance was considered at *P*<0.05. Adobe Photoshop was used to measure the size of cell wounds and for immunoblot images.
